# Thermal Conductivity and Mechanical Properties of Thermoplastic Polyurethane-/Silane-Modified Al_2_O_3_ Composite Fabricated via Melt Compounding

**DOI:** 10.3390/polym11071103

**Published:** 2019-06-29

**Authors:** Eyob Wondu, Zelalem Lule, Jooheon Kim

**Affiliations:** School of Chemical Engineering and Materials Science, Chung-Ang University, Seoul 156-756, Korea

**Keywords:** polyurethanes, thermal conductivity, mechanical properties, composites, extrusion

## Abstract

The increase of miniaturization and rise of powerhouses has caused a need for high-performing thermal interface materials (TIMs) that can transfer heat in electronic packaging. In this study, a thermoplastic polyurethane (PU)/alumina composite was produced via twin extrusion and was suggested as a TIM. The surfaces of the alumina particles were modified by γ-aminopropyltriethoxysilane (APTES) and then evaluated using Fourier transform infrared spectroscopy (FT-IR) and X-ray photoelectron spectroscopy (XPS). The field emission scanning electron microscopy (FE-SEM) images revealed that the addition of surface-modified alumina was well adhered in the PU matrix. The tensile strength of the composite remained unchanged, while the Young’s modulus showed improvement as compared to the pure PU. The elongation at the break decreased as the filler loading increased, due to the brittle behavior of the composite. The viscoelastic elastic property analysis results revealed that there was an increase in the storage modulus of the composite and the glass transition temperature curve shifted to the right. The thermal conductivity of the composite showed that there was an 80.6% improvement in thermal conductivity with the incorporation of 40% APTES-treated alumina particles.

## 1. Introduction

Power loss, small life span, and poor performance of electronic materials are observed when exposed to higher systems of temperatures, due to the creation of a large heat load from integrating many components in a large surface area-to-volume ratio. The increase of miniaturization and rise of powerhouses has caused a need for high-performing thermal interface materials (TIMs) that can transfer heat in electronic packaging [1,2,3,4]. Heat dissipation is necessary to appropriately operate electronic devices [1,2,4,5,6,7,8]. Many researchers have developed thermally conductive and electrically insulative composite materials as TIMs [2,6,7,9,10,11,12,13,14,15,16,17] Mostly, polymers are used as TIMs with their properties enhanced with the help of thermally conductive, less electrically conductive fillers. In this study, commercial-based thermoplastic polyurethane (PU), made from hard segments of methyl diphenyl diisocyanate and polyester-based soft segments, was used as a matrix. The polyols provide extensibility to the PU, while a high modulus is provided by the PU’s hard segments [3,4,18]. When used as a TIM alone, PU has a low thermal conductivity, 0.19 W/m.K. [19]; therefore, introducing thermally conductive filler materials to the PU matrix is a good option to enhance the thermal conductivity of the PU.

Three types of fillers can be used to improve the polymer matrix properties: Ceramic, metallic, and carbon-based. While metallic fillers improve thermal properties, they also enhance the electrical conductivity of the composite, which is undesirable in TIMs. Carbon-based fillers enhance the thermal conductivity of a matrix, provided that the mechanical properties and electrical conductivity [20], which are not desired in TIMs, are adequately controlled. Various ceramic fillers are used to fabricate thermally conductive composite materials because they are electrically resistive and influence the mechanical property of the matrix less negatively than other types of fillers. Examples of ceramic fillers include aluminum oxide, aluminum nitride, silica, montmorillonite, aluminum nitride, and boron nitride [1,4,17,21]. Ceramic fillers have the least effect on the electrical conductivity and mechanical properties and thus prevent power leakage; however, they often do not increase the thermal conductivity as high as metallic and carbon-based fillers, but their effect on mechanical and electrical property is less, compared to the other types. In this study, a ceramic filler, Al_2_O_3_, was used as a filler to enhance thermal and mechanical properties because of its low cost, availability, easy modification of its surface as compared to other ceramic fillers, high-temperature resistivity, and, most importantly, electrically insulating properties. A number of researchers have used aluminum oxide as a filler to enhance thermal conductivity of polymers to fabricate a composite used as TIMs [12,13,22,23,24].

To achieve high thermal conductivity, high loading of the Al_2_O_3_ filler, i.e., >50 volume %, is required, which can decrease the mechanical property performance of the composites and cause processing issues [4]. To address these problems, the filler is surface treated to decrease interfacial thermal resistance and enhance thermal conductivity of the filler [1]. Nonetheless, surface functionalization or surface improvement does not always lead to enhanced thermal conductivity due to defects, such as acoustic phonon transport [1]. Different surface improvement methods get applied to fillers to reduce interfacial thermal resistance, such as using surfactants [7], functional polymers [10], inorganic coatings [8,24,25,26,27], and coupling agents [16]. In this study, γ-aminopropyltriethoxysilane (APTES) was used as a coupling agent to modify the surface of the alumina particles. This study presumes that thermal conductivity is increased as the filler content increases, which weakens the composite’s mechanical properties unless the filler is fully dispersed to the polymer surface and attached to the matrix. As a result, appropriate filler loading with proper surface modification of the filler material is needed to maintain the mechanical property of the composite, while enhancing thermal conductivity. Kim et al. [2] used APTES-treated boron nitride (BN) to improve the thermo-mechanical property of PU and reported 0.6 W/(m.K) of thermal conductivity and a tensile strength increase from 2 to 3.3 MPa at a particle loading of 35% of APTES-treated BN. In addition, various researchers have also used APTES treatment for fabrication of composites used as TIMs [28,29].

We fabricated the PU composites, i.e., extruded with surface-modified alumina and pristine alumina, using a melt extrusion process. The thermal properties, mechanical properties, and morphology of the obtained products were characterized and evaluated using laser flash analysis (LFA) and thermogravimetric analysis, dynamic mechanical analysis (DMA) and universal testing machine (UTM), and field emission scanning electron microscopy (FE-SEM), respectively.

## 2. Materials and Methods

### 2.1. Materials

Alumina particles (Al_2_O_3_) with average diameters of 10 μm were obtained from Sigma Aldrich, St. Louis, MI, USA and used as a filler for the matrix. The polyester-based pellet-type thermoplastic polyurethane (PU) was obtained from Dongsung Chemical, Ulsan, Korea and used as a matrix. The γ-Aminopropyltriethoxysilane (APTES), used to modify the alumina particle surfaces, was obtained from Alfa Aesar (Ward Hill, MA, USA) and used without further processing. Potassium hydroxide (KOH) and ethanol, respectively used for hydroxyl treatment and as a solvent for surface modification of the alumina particles, were obtained from Dae-Jung Chemical and Metal Co. Ltd. (Dae-Jung, Korea).

### 2.2. Methods

The surface of the filler was treated with APTES (aminopropyltriethoxysilane) as follows: The alumina was first OH-treated using 0.5 M KOH solution in 300 mL DI (deionized) water. The OH-treated alumina was washed using a vacuum filter several times until the base was neutralized. Once the base was neutralized, the alumina was oven-dried for 24 h at 80 °C. The dried alumina was treated with 2% by mass of alumina APTES, using ethanol as a solvent for 23 h with a magnetic stirrer and condenser hold to trap the distilled ethanol at 80 °C. The APTES-treated alumina was washed twice in a vacuum filter and oven-dried for 24 h at 80 °C. The dried alumina was used as a filler for comparison with pristine alumina in the polyurethane matrix to form a polyurethane composite for electronic packaging.

**Figure 1:**
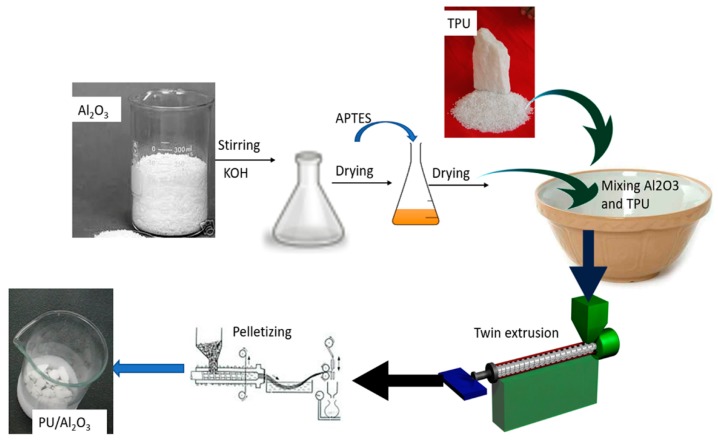
Scheme for production of pure polyurethane (PU)/Al_2_O_3_ composites.

The thermoplastic polyurethane and surface-treated alumina were dried at 60 °C for 24 h prior to fabrication of the composite to avoid void formation in case there was absorbed moisture. The polyurethane composite with polyurethane as a matrix and surface-modified alumina as a filler was produced in a twin extruder (model BA-11, L/D ratio = 40, Bau Technology, Seoul, Republic of Korea) at a processing temperature of 215 °C and an extrusion speed held at 70 rpm. The extruded composite was pelletized and oven-dried at 60 °C for 24 h. Specimens for other tests were prepared by molding the extrudate in a compression molding machine (model BA-915, Bau Technology, Seoul, Republic of Korea). The molded mixture was cooled to room temperature and the cooled specimen was placed in an oven at 80 °C for at least 24 h before any characterization was performed to remove absorption of any moisture. The formed composites were polyurethane/20% treated alumina, polyurethane/30% treated alumina, polyurethane/40% treated alumina, polyurethane/40% pristine alumina, and pure polyurethane, which we abbreviate as 20PU, 30PU, 40PU, 40PPU, and PU, respectively. All percentages are weight percentages (wt/wt %). The scheme used while producing the composite is shown in Figure 1.

### 2.3. Characterization

The chemisorption of APTES into the alumina particles was analyzed through Fourier transform infrared spectroscopy (FT-IR; Nicolet, is5, Thermo Fisher Scientific, Seoul, Korea) and X-ray photoelectron spectroscopy (XPS; K-Alpha, Thermo Fisher Scientific). During FT-IR analyses, KBr (potassium bromide) powder was used as the background for the spectrum and data were recorded within a frequency range of 4000–400 cm^−1^. For XPS measurements, a survey pass energy of 200 eV and a step size of 1.0 eV were used. Monochromatic Al Kα was applied as the background and the obtained result was curve-fitted, provided that all the Gaussian-fitted width peaks of the spectrum were held constant.

The adhering property of alumina particles to the PU surface (morphology) was analyzed after fracturing all the specimens by liquid nitrogen, using Field Emission Scanning Electron Microscopy (FE-SEM; Sigma, Carl Zeiss, Oberkochen, Germany) after coating all specimen layers with platinum to inhibit accumulation of charges.

The thermal stability of both the surface-modified alumina and PU/alumina composites were studied using thermogravimetric analysis (TGA). About 10 mg of each sample was taken and heated to a temperature of 600 °C at a heating rate of 10 °C/min to check the thermal degradation in an air atmosphere. Laser Flash Analysis (LFA467 Hyper-Flash, Netzsch Instrument Co., Selb, Germany) was used to measure the thermal diffusivity, δ (mm^2^/s), of each composite at room temperature. The density, ρ (g/cm^3^), of each specimen was calculated using the rule of mixture, and the specific heat, C_P_ (J/ (g.K)), was determined using the differential scanning calorimetry (DSC, DSC-7, PerkinElmer Co., Mougins, France); and then the thermal conductivity was calculated.

The dynamic mechanical properties of the PU, 20PU, 30PU, 40PU, and 40PPU composites were measured using dynamic mechanical analysis (DMA; Triton Tech., London, UK) in the two-point bending mode at a frequency and temperature range of 1 Hz and −60 to 160 °C, respectively. The tensile properties of all composites were analyzed using a Universal Testing Machine (UTM; model UTM-301, R&B Corp., Daejeon, Korea) at room temperature with a crosshead speed of 5 mm/min, tension loading rate of 10 mm/min, and load force of 100 Kg forces per millimeter. Four sample tests were performed for each composite and the average value was taken for the tensile strength, Young’s modulus, and elongation at break determination. The specimens were prepared according to the American Standards Testing Method (ASTM) specification D412.

## 3. Results and Discussion

### 3.1. Surface Functionalization

**Figure 2:**
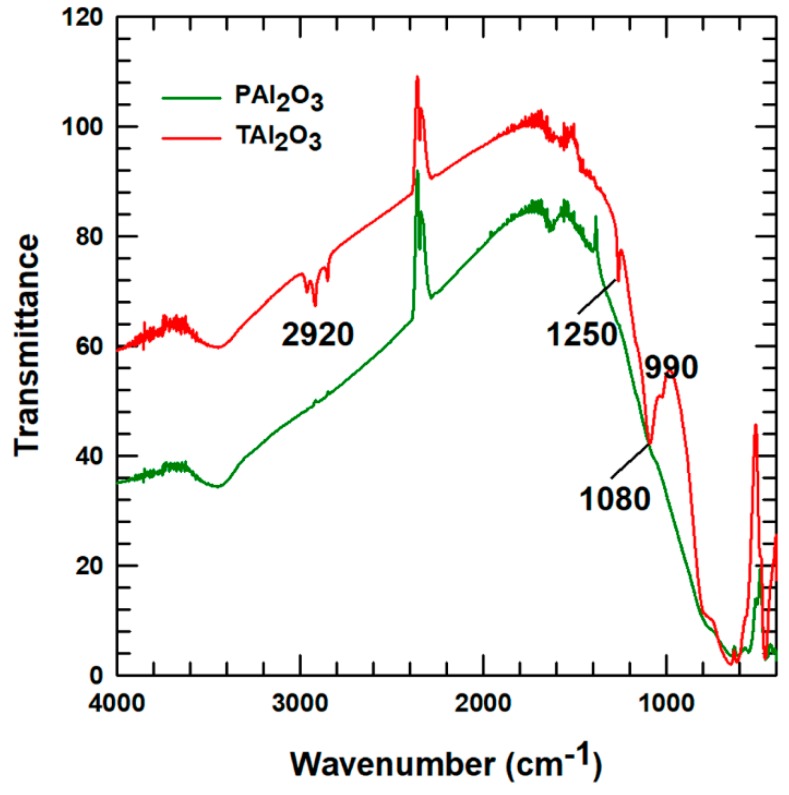
Fourier transform infrared spectroscopy (FT-IR) results for alumina particles used in this study: Pristine alumina (PAl_2_O_3_) and alumina treated with γ-aminopropyltriethoxysilane (APTES) (TAl_2_O_3_).

The alumina was characterized to verify the attachment of APTES, as compared to the pristine alumina. The FT-IR curves for the alumina particles were evaluated, as shown in Figure 2. Hydroxyl group peaks were found between 3600 and 3000 cm^−1^. The dark green line in Figure 2 shows how the APTES-treated alumina curve has new peaks that are not present in the pristine alumina curve. The peaks observed at wave numbers between 2900 and 2860 cm^−1^ correspond to –CH_2_–, 1250 cm^−1^ is due to SiO–CH_2_ bonds of the silane groups attached to the alumina particles, and 1080 cm^−1^ corresponds to NH_2_ of the amine group from APTES. Moreover, the peak at wavenumber 990 cm^−1^ is the binding peak for Si–O–R [2,11]. Therefore, the new peaks observed for SiO–CH_2_, NH_2_, and Si–O–R in the FT-IR curve suggest that the alumina particles were surface-improved with the addition of APTES.

**Figure 3:**
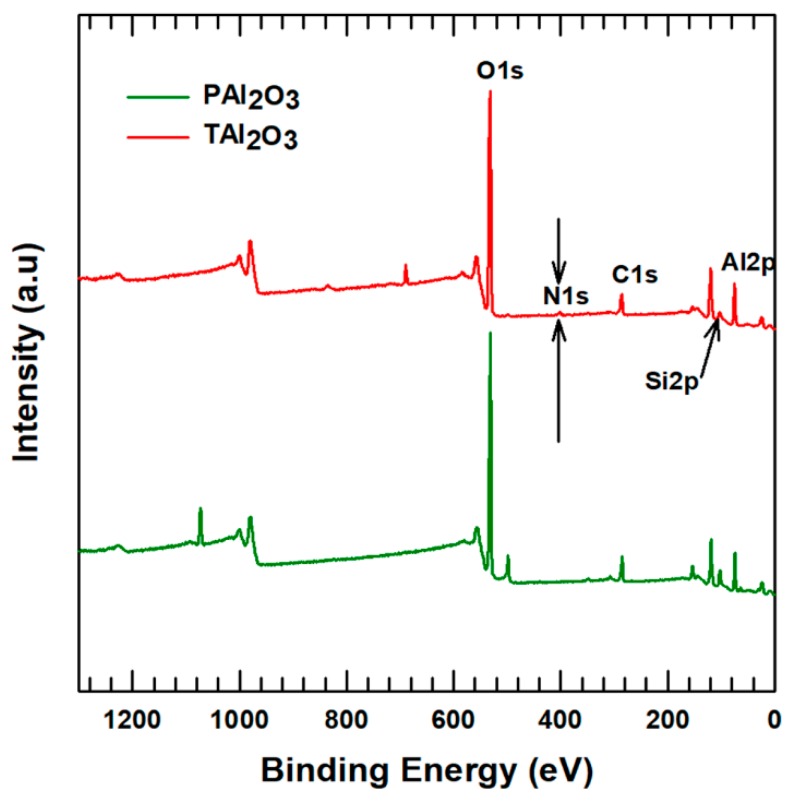
**Figure 3**. X-ray photoelectron spectroscopy (XPS) results of pristine alumina (PAl_2_O_3_) and treated alumina (TAl_2_O_3_).

XPS was employed to analyze the elemental composition of the surface-modified alumina compared to the pristine alumina. The scan surveys for the surface-modified alumina and pristine alumina are shown in Figure 3. The results of XPS show that N, C, Al, Si, and O are existing on the treated alumina surface, while only C, Al, and O are present in pristine alumina. The surface-modified alumina has formed a new peak, the N1s peak has been increased, and the other peaks either increased in peak height or decreased. The intensity of the Al2p peaks dropped by 7.95% and the intensity of C1s increased by 3.69% after treating the alumina surface with APTES. The increase in the intensity of C1s is small because the APTES amount added is only 2% of the total alumina, however, the increase in its intensity shows that the APTES has functionalized with the surface of alumina particles.

**Figure 4:**
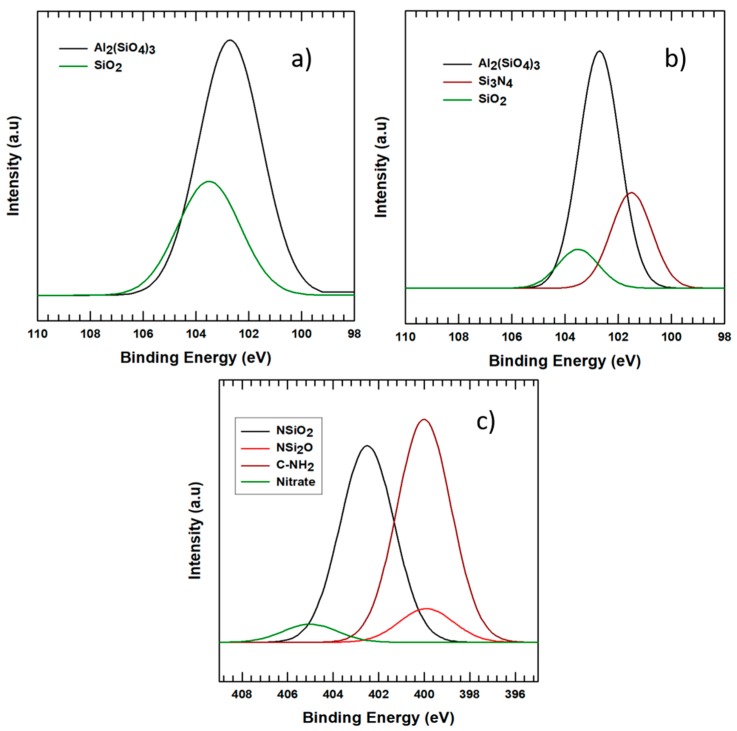
XPS deconvolution results for (**a**) silane-treated Si2p peaks, (**b**) pristine alumina Si2p, and (**c**) silane-treated N1s peaks.

To further analyze the bonds, deconvolution was performed for both the surface modified and pristine alumina. The deconvolution results for Si2p are shown in Figure 4a,b, respectively, for the surface-modified alumina and pristine alumina. Figure 4c shows the deconvolution results of N1s. The Si2p curve of APTES-treated alumina peaks was resolved into two as shown in Figure 4a, while it was resolved into three peaks for the pristine alumina (Figure 4b). Even though the number of peaks are reduced in the APTES-treated alumina, the peak intensity for SiO_2_ peaks appearing at a binding energy of 103.5 is increased as compared to the pristine alumina. The peak intensity for Al_2_(SiO_4_)_3_ appearing at a binding energy of 102.7 eV also increased, but the peaks for Si_3_N_4_ disappeared in the surface-modified alumina and this may be due to the incorporation of amide bonds into C–NH_2_ in N1s peaks. The increase in peak intensity shows the attachment of APTES to the surface of alumina particles. As shown in Figure 4c, the N1s peaks have resolved into four different peaks. There is no new peak appeared in the pristine alumina, which shows that there are no Nitrogen groups in the pristine alumina. The deconvoluted N1s peak on the treated alumina curve has a new peak intensity of 405 eV for nitrates, 402.5 eV for NSiO_2_, and 399.9 and 400 eV for NSi_2_O and C–NH_2_, respectively. The C–NH_2_ peak is caused by the bond between carbon and nitrogen atoms that are originating from the connection of APTES and alumina particles. Therefore, the appearance of N1s groups together with an increase in the peaks of Si2p peaks on the APTES-treated alumina particles shows attachment of APTES on the surface of alumina.

### 3.2. Morphology

**Figure 5:**
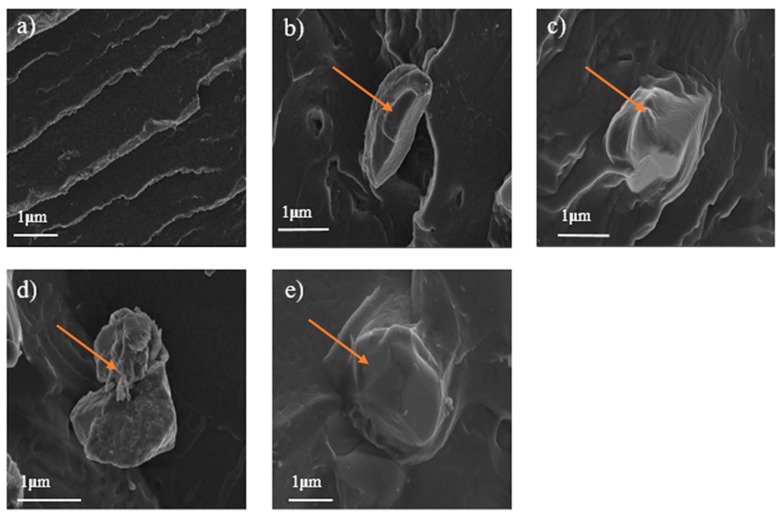
SEM images of PU composites: (**a**) PU, (**b**) 20PU, (**c**) 30PU, (**d**) 40PPU, and (**e**) 40PU.

To investigate the distribution of Al_2_O_3_ in thermoplastic polyurethane composites, the morphologies of the pure PU and PU/alumina composites were observed using FE-SEM. The FE-SEM micrograph images for the PU, 20PU, 30PU, 40PPU, and 40PU specimens at a magnification of 5000× and 1 µm are shown in Figure 5a–e, respectively. The images of samples after a surface-modified filler addition show morphological differences in the PU matrix. The arrow on the images shows the alumina particles. Figure 5c,e illustrates that the alumina particles adhered to the PU matrix and the composite became more brittle, which is associated with smooth surface properties after the addition of surface-modified alumina particles. In addition, pure PU has a uniform rough fracture surface morphology (Figure 5a), which might be due to more ductile properties compared to the PU composite (Figure 5e). Figure 5b indicates that there is no adherence between the PU matrix and alumina particles, due to the low size of alumina particles, 20%. As shown in Figure 5d, the alumina particle did not adhere to the 40PPU surface; it appears just on the surface, which is due to the surface non-functionalization of alumina particles.

### 3.3. Thermal Degradation and Thermal Conductivity

**Figure 6:**
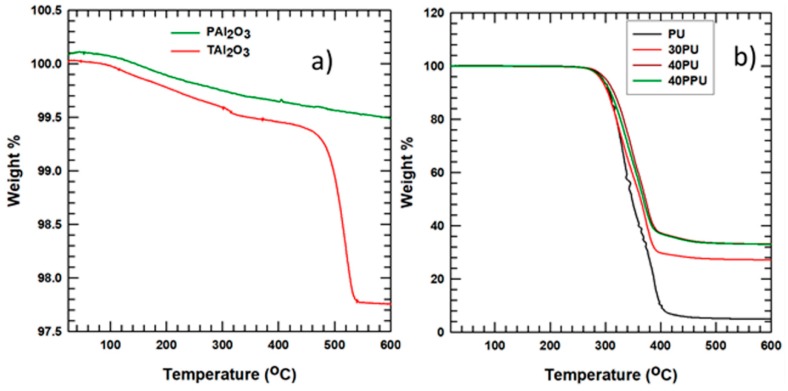
Thermogravimetric analysis (TGA) results for (**a**) pristine alumina (PAl_2_O_3_) and surface-modified alumina with 2% APTES (TAl_2_O_3_) and (**b**) composite PU samples.

The thermal degradation properties of Al_2_O_3_ and APTES-treated alumina (Al_2_O_3_) were analyzed using TGA by heating in air at 600 °C, as shown in Figure 6a. The mass of the alumina decreased by 2.3% for an APTES weight percent of 2%. This indicates that the APTES modified the surface of alumina particles, as it increased the weight of alumina by 2%.

The composites’ thermal degradation properties were analyzed at 600 °C for PU, 20PU, 30PU, 40PU, and 40PPU by heating with air. The TGA curve for the composites is shown in Figure 6b. From the graph, it is clear that the alumina was attached to the matrix for all composites, as the alumina mass percentage appears even at 600 °C, and the polymer got degraded at a temperature of around 410 °C. Almost all PU weights degraded at temperatures of around 410 °C, although the PU/alumina composite had different thermal degradation properties, indicating that the ceramic is resistant to heat.

**Figure 7:**
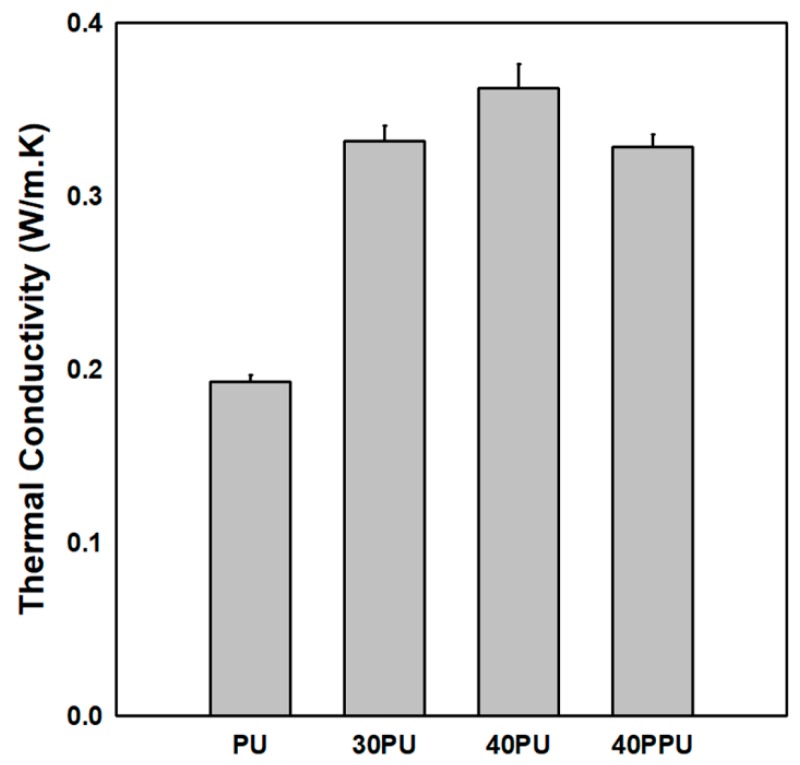
Thermal conductivity of PU and PU/Al_2_O_3_ composites.

Thermal conductivity can be measured using steady-state methods, i.e., when stability has been achieved in the system, and transient methods, i.e., used while heating or cooling a material. Steady-state methods include a guarded hot plate, axial flow, heat flow, and pipe methods. Transient methods, including the flash, transient hot wire, and transient plane source, measure thermal diffusivity [1]. In this study, the thermal conductivity of a composite was calculated after measuring the thermal diffusivity of the specimens using the laser flash analysis (LFA) transient method, provided that each specimen was prepared to a size of 1 cm^2^. Here, thermal conductivity was calculated as follows:

(1) $$Ƙ=\delta \rho C_{p}$$

where Ƙ is thermal conductivity, δ is thermal diffusivity, ρ is density, and C_p_ is specific heat. The results obtained from the LFA analysis are shown in Figure 7, which indicate that increasing the percentage of filler loading increased the thermal conductivity of the composite. This increase was due to the high filler to filler interactions in the composite, which in turn increased the composites’ thermal conductivity. The thermal conductivity of the 40PU was 80.6% higher than that of the PU and 10.6% higher than that of the 40PPU; these higher values were due to the surface modification of alumina by the APTES, which created strong adhesion between the alumina and PU.

### 3.4. Viscoelastic Properties

**Figure 8:**
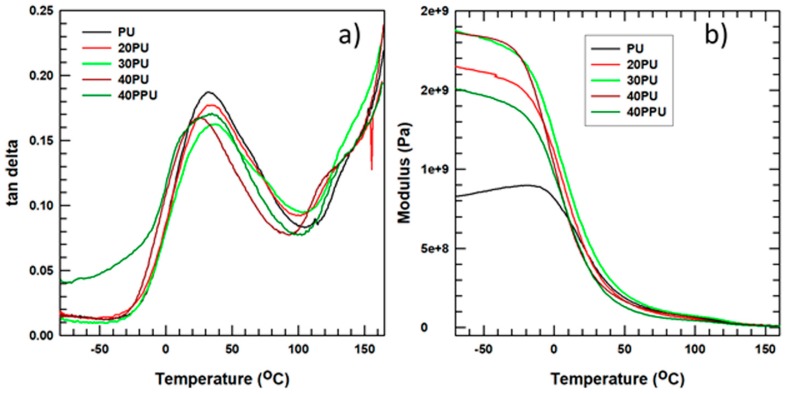
Dynamic mechanical analysis (DMA) results for PU samples: (**a**) Tan delta curve and (**b**) storage modulus curve.

An increase in filler loading affects both the mechanical and thermal properties of the composite. Dynamic mechanical analysis (DMA) was used to investigate the effect of filler loading on the viscoelastic properties (storage modulus and tan δ peak) of the composite created in this study. In the tan δ peak (Figure 8a), there is a decrease in the peak height and a shift to the right position (higher temperature) of the glass transition temperature (T_g_). The decrease in peak height is observed for the PU/surface-modified alumina composite, compared to the polyurethane/pristine alumina composite, due to the incorporation of the surface-modified filler particles in the polyurethane matrix. The good dispersion of alumina particles [6] in the matrix (PU) inhibited the mobility of the PU chain, which improved the mechanical properties for polyurethane/surface-modified alumina composites.

Figure 8b shows the storage modulus of the composite, which is a measure of the stored energy, and the PU as a function of temperature. The curve shows that the storage modulus increased from 8.12 × 10^8^ to 1.92 × 10^9^ Pa, a 132% increase, as the surface-treated alumina content increased from 0% to 40% before the glass transition temperature was reached. This improvement was due to the reinforcement gained by the interaction between the APTES and alumina, which in turn caused a decrease in the slope of the curve that was observed near the glass transition temperature, due to the relaxation of the polymer chains.

### 3.5. Tensile Properties

**Figure 9:**
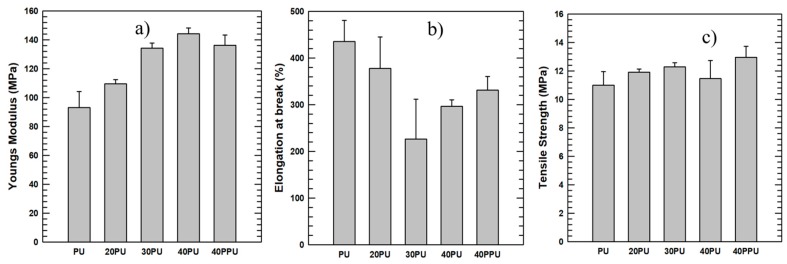
Tensile properties of PU samples: (**a**) Young’s modulus diagram, (**b**) elongation at the break, and (**c**) tensile strength.

The tensile characteristics of the pure PU and composite PU/alumina samples were measured with a UTM machine at room temperature. The composites’ tensile test results are shown in Figure 9. The stress–strain curve slope is the Young’s modulus, which is a measure of the resistance to elastic deformation of a material and reflects stiffness of a material. A material with a high Young’s modulus is stiff and high loads are required for deforming such materials, and vice versa. Similar to the storage modulus, the stress–strain diagram slope showed an increase in the Young’s modulus, which indicates that the composite became stiffer than the pure PU. Figure 9a shows the 40PU with a modulus of around 143 MPa, while pure PU has a modulus of around 91 MPa. In addition, compared to the 40PPU, the 40PU alumina has a higher Young’s modulus and is thus stiffer. Therefore, modifying the surface of the alumina filler before fabricating the composite improved the stiffness of the composite. We also deduced that increasing the filler percentage also increased the Young’s modulus, which agrees with the results obtained by various researchers [2,3,22,23] .

The elongation at the break of the composite as a function of alumina loading percentage is shown in Figure 9b. The figure illustrates that an increase in filler loading decreased the elongation at the break, which was due to the incorporation of alumina particles in the PU matrix. The result shows that there is a change in the PU matrix from a ductile material to a more brittle composite material, which agrees with the result shown in morphology analysis. The elongation at the break of 30PU is the lowest as compared to the others; nevertheless, the result shows it is applicable in various applications that require high stiff and brittle composite materials. The 40PU’s elongation at the break is somewhat less than that of 40PPU, but this result is compensated by the stiffness of the 40PU, which has a 12.65% higher modulus than that of 40PPU.

The tensile strength of thermoplastic polyurethane/alumina composite is shown in Figure 9c, obtained from the stress–strain graph. The tensile strength of the composite appears to be almost the same as the pure PU, although a slight increase in tensile strength is observed with increasing filler content. This is due to the advantage gained from highly stiff and high tensile strength Al_2_O_3_ particles, which makes the polymer composite more resistant to low tensions, as low as 10MPa, contrary to other researchers’ works [30,31], which show a decrease in tensile strength. In summary, the addition of highly stiff alumina particles to the thermoplastic polyurethane and attachment of alumina particles affected the mechanical properties of the composite.

## 4. Conclusions

Composite samples of PU with alumina were prepared and their mechanical and thermal properties were compared. We found that the thermal conductivity of the composite PU improved by 80.6% compared to pure PU extruded with 40% by mass APTES-treated alumina. The glass transition temperature of the pure PU and the composites prepared from it with alumina were almost identical. The adherence properties of the matrix with the filler were studied using FE-SEM. The result revealed strong adhesion when using the surface modified alumina. The stiffness values of the composite PUs were greater than those of the pure PU; the 40PU had 55% more Young’s modulus and 132% more storage modulus than the pure thermoplastic polyurethane. However, the tensile strength of the composite remained almost constant as compared to that of the pure PU. The storage modulus increased by 22.53% for the 40PU compared to that of the 40PPU. Therefore, in summary, an 80.6% increase in thermal conductivity, a 55% improvement in Young’s modulus, and 132% increase in storage modulus were achieved with the composite as compared to the PU. The thermal conductivity and Young’s modulus of the 40PU were respectively 10.6% and 12.65% higher than that of the 40PPU.
